# Development of a community-based peer-support intervention to improve contraceptive agency and diffuse self-injectable contraception in Uganda: Application of the human-centered design approach

**DOI:** 10.1186/s12905-025-03614-5

**Published:** 2025-03-10

**Authors:** C Birabwa, B Phillips, D Amongin, L Atuyambe, A Turinayo, J Etap, B Kaudha, S Alum, K Agnes, J Kramer, E Sedlander, J Liu, K Holt, P Waiswa

**Affiliations:** 1 Department of Health Policy, Planning and Management, Makerere University School of Public Health, Kampala, Uganda; 2 Department of Family and Community Medicine, School of Medicine, University of California, San Francisco, United States; 3 Department of Community Health and Behavioural Sciences, Makerere University School of Public Health, Kampala, Uganda; 4 Design Without Borders, Kampala, Uganda; 5 Department of Mechanical Engineering, University of Michigan, Ann Arbor, United States; 6 Institute for Health and Aging, School of Nursing, University of California, Oakland, United States; 7 Global Public Health, Karolinska Institute, Stockholm, Sweden; 8 Busoga Health Forum, Jinja, Uganda

**Keywords:** Human-centered design, Contraceptive agency, DMPA-SC self-injection, Uganda, Peers, Contraception, Intervention design, Social support

## Abstract

**Background:**

The low use of self-injectable contraception, coupled with the recognition that many individuals need support beyond training to use self-care technologies successfully, suggests the need for innovative programming. We describe the participatory human-centered design process we used in two districts of Uganda to develop a community-based peer support intervention to improve women’s agency to make and act on contraceptive decisions and help diffuse self-injectable contraception.

**Methods:**

The design team included multi-disciplinary researchers from Uganda and the United States, representatives of local community-based organizations and village health teams, and local women of reproductive age. The research group conducted 21 in-depth interviews, 12 observations, and six focus group discussions to understand women’s social support needs, contraceptive-seeking experiences, and communication channels. From these data, the design team derived insights into needs and opportunities to improve contraceptive agency and support self-injection use among interested women, spurring a creative idea-generation process to develop a large set of potential solutions. We collectively prioritized the most promising ideas into an integrated, theoretically informed intervention and subsequently prototyped, tested, and refined the intervention.

**Results:**

Design insights included: women value information from experienced peers and want support to navigate uneven partner dynamics, conflicting contraceptive information, concerns about contraceptive-related side effects, and unreliable contraception services. The final intervention—called *I-CAN *(English)*, Nsobola* (Lusoga)*, An Atwero *(Langi)—engages experienced self-injection users as ‘mentors’ to support other women (‘mentees’) they recruit in community-based settings. Mentors provide informational, instrumental, appraisal, and emotional support tailored to the individual needs of mentees. This support is designed to improve mentees’ knowledge, consciousness of their rights related to contraception, self-efficacy, and perceived control related to contraceptive decision-making, self-injection self-efficacy, contraceptive access, and ability to act on preferences.

**Conclusions:**

Our iterative human-centered design process incorporated diverse, lived experiences and scientific expertise and resulted in a peer support intervention with the potential to fill an important gap in contraception service delivery in Uganda. Our approach demonstrates that creating complex interventions to prioritize support for women’s agency related to contraception in line with a human rights-based approach and to spread new contraceptive methods is feasible.

**Supplementary Information:**

The online version contains supplementary material available at 10.1186/s12905-025-03614-5.

## Background

Self-injectable contraceptives have been lauded for their “empowering” potential [1, 2]. Yet, uptake of self-injection (SI) of subcutaneous depot medroxyprogesterone acetate (DMPA-SC) has been slow globally. For example, in Uganda, only 8% of DMPA-SC users self-inject despite the option being introduced in 2017 [3]. Recent research suggests that the conventional approach to introducing and scaling self-injection through healthcare provider training and “demand creation” strategies[4–10] is limited in effectively reaching those who may benefit most from this self-care technology's unique potential [11–14]. Notably, many women, including in Uganda, fear injecting themselves even when they know about and are theoretically interested in the option [13–15]. This highlights the need to design SI programs to meet women’s needs and preferences for support within and outside the formal healthcare system [4, 16]. Further, to ensure a human rights-based approach to contraception programming, SI and other single method-focused contraception programs should support women to have agency in making their own contraceptive decisions, which may include choosing not to use a contraceptive or choosing to use a method *different* from the particular method the program is focused on promoting [17, 18].

Peer support has been highlighted as a promising way to support contraceptive self-injection use and could also be leveraged to support contraceptive decision-making more broadly [6, 19, 20]. Systematic reviews have documented the promise of peer support interventions across many areas of health, particularly for rural or other populations not well reached by the healthcare system [21–23]. Peer support related to contraception includes information sharing, instrumental support to overcome barriers, and emotional or appraisal support to help solve problems or overcome fears. Despite its promise for contraception, evidence-based approaches to leveraging peer social support are inadequate. When mobilizing peers to support women to make and act on their own contraceptive decisions, it is important to acknowledge existing literature, which shows that peers can hinder women’s agency to make contraception decisions when they share incorrect information [24–28]. For example, a recent socio-centric network study from Kenya found the belief that ‘contraceptives cause infertility’ was associated with lower odds of contraceptive use among women whose peers held this belief [24].

This paper delineates the design activities we conducted and outputs we generated using a human-centered design (HCD) approach to develop a community-based peer social support intervention to increase women’s contraceptive agency and support women to successfully self-inject with DMPA-SC if they choose this method in Mayuge and Oyam districts in Uganda.

## Methods

### Design approach and theoretical frameworks

#### Human-centered design

HCD is a creative approach to problem definition and solution development that engages stakeholders in the design process [29]. Applications of HCD to address public health challenges are gaining prominence, including in Uganda [29–33]. We used HCD to develop a deep understanding of key stakeholders, including women using or potentially interested in self-injecting with DMPA-SC. This ‘understanding’ included women’s self-defined contraceptive needs, preferences, and critical contextual factors influencing their agency and awareness or use of SI. We harnessed this empathetic understanding and active engagement of community members to develop solutions that meet their needs [29, 30].

**Fig. 1:**
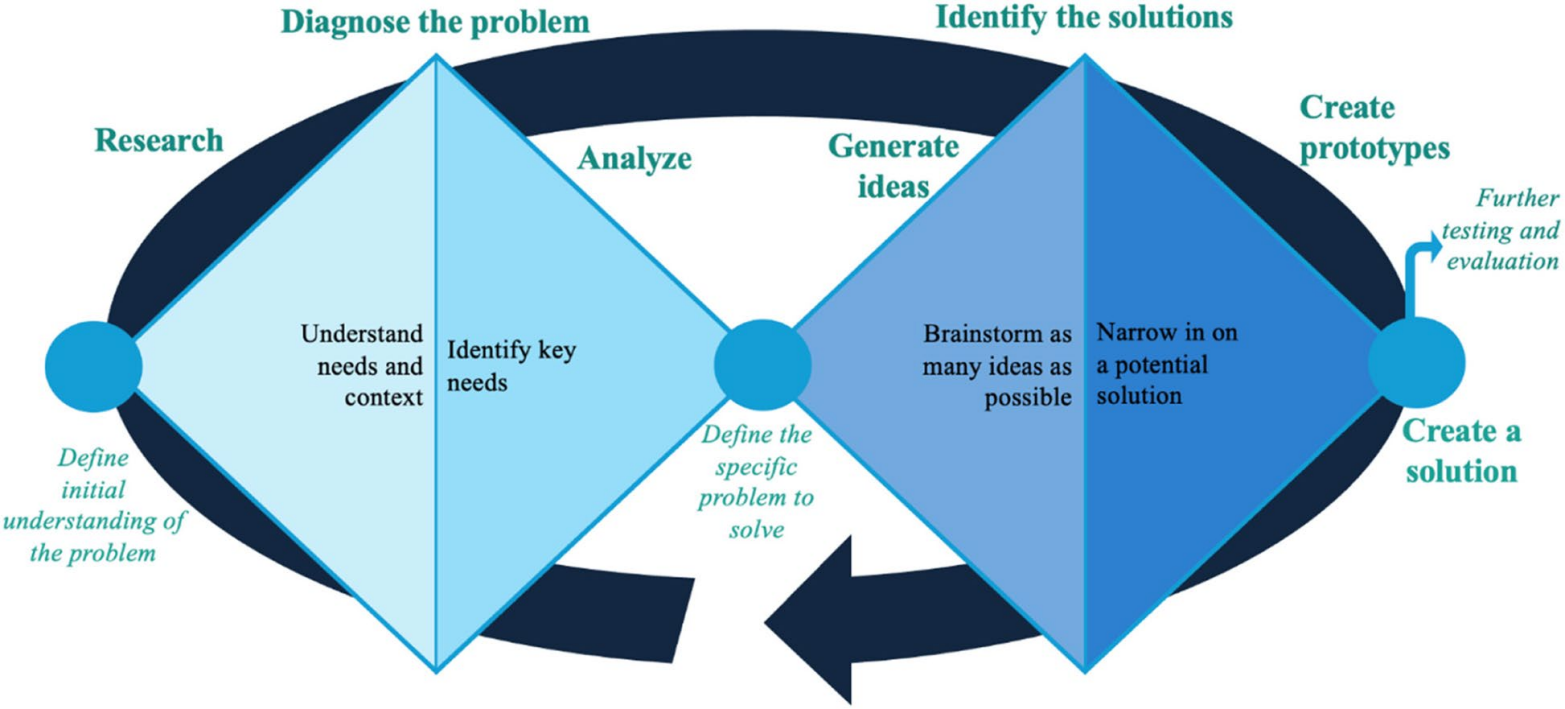
Double Diamond approach (Image adapted from UK Design Council double diamond; labels added by authorship team) [34]

Inspired by the “double diamond” model, we structured our HCD process in two cycles (Fig. 1) [34]. In Cycle 1, we diagnosed the problem to be solved. This involved an initial divergent process fostered by empathy and curiosity to investigate women’s experiences in making or acting on contraception decisions. This process enabled us to identify the root causes of women's barriers and develop insight statements guiding subsequent processes. In Cycle 2, we identified solutions to address the priority barrier(s) defined in Cycle 1. Cycle 2 began with a divergent process to develop solution ideas, followed by a convergent process to refine and narrow down the solutions to those most likely to address user needs. Testing and feedback loops were integrated, ensuring the selected solutions fit our working context.

#### Theoretical frameworks

Our design process was undergirded by two theoretical frameworks. First, we used the Diffusion of Innovations (DOI) theory, which describes how new technologies are adopted in populations, [35] to guide intervention component selection. DOI constructs include *observability,* which suggests that self-injection spread will occur when women observe self-injection among other women, and *relative advantage,* which suggests women will use self-injection if there is a relative advantage that they can take advantage of compared to other contraceptive methods. Second, we used the Contraceptive Agency Framework, which our team recently published, drawing on multidisciplinary agency and health behavior theories to delineate psychosocial constructs necessary for individuals to have agency over what, if anything, they do to avoid pregnancy when they are not actively seeking pregnancy [36]. This framework is rooted in human rights principles. It helps operationalize calls to design and evaluate contraceptive programs not with contraceptive *uptake* as the end goal but rather to support women’s right to make and act on *their own decisions* related to contraception [37–40].

### Design setting

Our design process was set in Mayuge (Baitambogwe sub-county) and Oyam (Loro sub-county) districts from Eastern and Northern Uganda, respectively. We purposefully selected these districts because DMPA-SC had been available in public health facilities for at least one year; partner and Ministry of Health support for DMPA-SC stock and provider training in self-injection of DMPA-SC existed at the time; and contraceptive prevalence rates were relatively low in 2021 when our design work began, indicating that women still face barriers to using contraception when they so desire. While they vary geographically, historically, and socio-culturally, the districts are primarily rural, with one-third of women of reproductive age (WRA) reportedly using contraception [41].

### Design team

Our design team included two groups: 1) a research group and 2) design community advisory boards (dCABs) in each district. The research group was multidisciplinary and comprised researchers from Uganda and the United States with engineering, public health, medicine, and social and behavioral sciences backgrounds. The dCABs comprised representatives from two community-based organizations (CBOs) who would eventually implement the final intervention and female community members, and village health team members purposely selected by CBOs to represent diverse socio-economic profiles and contraception experiences.

### Design Process

This section describes our main activities during each design process cycle, as depicted in Fig. 1. Cycle 1 (diagnose the problem) included initial research and analysis to deepen our knowledge of target users’ needs, challenges, and future desires. In **Cycle 2** (identify solutions), solutions were iteratively generated, tested, and refined into a holistic intervention to implement. All materials, including instruction manuals, interview guides, and PowerPoint slides, were translated into the local languages (Langi and Lusoga) before each design workshop. The workshops were conducted primarily in Langi and Lusoga, with English, Luganda, and Acholi as secondary languages. Drawing on their lived experiences, dCAB members shared their views on proposed design questions, validated research findings, and proposed candidate solutions. Facilitated by design consultants, the research group participated in in-person and virtual interactive design workshops with the dCABs at different stages of the design process.

## Diagnose the problem

### Initial research

We conducted research with WRA (aged 15–45 years), community health workers (members of Village Health Teams or VHTs), and health facility-based health workers (nurses and midwives) in November and December 2021. WRA included users and non-users of contraceptives and leaders of local women's groups, such as saving groups. We used informal referrals to identify current and potential future SI users and VHTs with experience in training women to self-inject. A team of four trained female research assistants recruited, consented, and interviewed participants. Participants were verbally consented and compensated with 30,000 Ugandan Shillings (about $8 U.S. dollars).

We collected data through in-depth interviews (IDIs), focus group discussions (FGDs), and structured observations. IDIs with women (*n* = 21) followed an interview guide with probing for emergent issues to understand women’s preferences with fertility management, contraceptive decision-making, and the types of people and places they used or preferred for contraceptive services and social support. Interviews lasted about an hour and were audio recorded with the respondent’s permission. We also interviewed two VHTs and one midwife using a semi-structured interview guide about their perspectives on training women to self-inject with DMPA-SC, challenges faced with contraceptive provision and counseling, including self-injection of DMPA-SC, and potential solutions to help reach women who desire to self-inject with DMPA-SC. We facilitated FGDs (*n* = 6 groups, with 6–8 participants per group) using a topic guide with adolescents, women’s group leaders, and married contraceptive users to deepen our understanding of women’s contraceptive decision-making and to explore how gender and social norms arise and diffuse amongst peers in the respective communities. To empathize with target users' and other stakeholders' daily lived experiences, we conducted structured observations (*n* = 12) for 5–8 h among selected women and VHTs we interviewed. While shadowing the participants, we noted what the women did, whom they interacted with, what challenges they experienced, what decisions they faced and made, and what influenced their choices. Table 1 shows the number of participants organized by data collection method.

**Table 1:** Participants by data collection method in human-centered design research

**Respondent category**	**Data collection method**					
	**IDIs (n of women interviewed)**		**FGDs (n of groups)**^**a**^		**Observations (n of women observed)**	
	**Mayuge**	**Oyam**	**Mayuge**	**Oyam**	**Mayuge**	**Oyam**
Users and non-users of contraceptive methods	7	7	2	2	3	2
Women group leaders	2	2	1	1	1	1
Village health team (VHTs)	0	2	0	0	1	2
Healthcare providers (midwives, nurses)	1	0	0	0	1	1
**Total**	**21**		**6**		**12**	

^a^ each group consisted of 6–8 participants each

### Analysis

We organized the research data using affinity mapping on *Miro*, a collaborative online data visualization platform. First, we reviewed the data, pulled out excerpts relevant to our design challenge (to support women’s contraceptive decision-making and desired self-injection with DMPA-SC), and gathered the excerpts into similar groupings. Second, as more data points were added and grouped, we identified themes, merged similar groups, and tweaked the phrasing of each group’s thematic title, considering their relevance to exposing the needs and opportunities to address our design challenge. Third, we developed initial insights by rephrasing the themes as statements articulating motivations for women’s contraceptive decisions and potential areas for solutions (insight statements). We refined the insights to specify women’s contraceptive needs, existing challenges, and solutions opportunities. We prioritized insights that aligned with our goal to support women’s contraceptive decision-making and self-injection of DMPA-SC among women who choose it.

In light of our insight statements and to better articulate our target users, we created personas (archetypes of women who could benefit from our solution), ecosystem maps (representations of crucial factors that influence target users), and journey maps (snapshots of women’s current life experiences highlighting key daily activities, goals, challenges, and points of possible intervention) [42]. The personas and journey maps were leveraged throughout the design process to remind team members of the specific experiences of those who may eventually benefit from our future intervention.

## Identify solutions

### Generate Ideas

We reframed our insight statements into ‘how might we’ (HMW) prompts to guide the design team in idea generation. HMWs are concise statements that innovate on specific challenges and opportunities found during the research stage while keeping the target users’ needs at the center [43]. We used two HMW prompts: 1) *how might we encourage/equip social groups with factual and relevant family planning information to support women in making and acting on contraceptive decisions, including self-injecting a contraceptive?* 2) *how might we communicate the availability of contraceptive methods at various access points so that all women can have consistent access to contraception, including self-injectable contraceptives?* We specified the key features that desirable ideas needed to have: 1) the idea involves supporting agency in women’s contraceptive decision-making, regardless of what they choose, recognizing that some women will make an informed choice not to use contraception; 2) the idea provides support for SI with DMPA-SC not currently available in the formal healthcare system; and 3) the idea includes some element of peer support or communication.

Using the HMWs and drawing upon the journey maps, ecosystems, and personas, dCABs brainstormed over 90 ideas to address the needs framed in the HMWs. Then, they engaged in open discussion to rank and vote on the ideas that fulfilled all three features, helping us to reduce the list of ideas to 24. The research group further evaluated these 24 ideas to narrow them into a smaller, coherent set of three solution ideas, looking at the following criteria: *relevance* to the design challenge, *feasibility* (an idea could be piloted within the project’s budget, and timeframe, and with an eye towards longer-term sustainability); and *desirability* (idea addresses the fundamental needs, challenges or opportunities elucidated in the design research).

### Create prototypes and test solutions

**Fig. 2:**
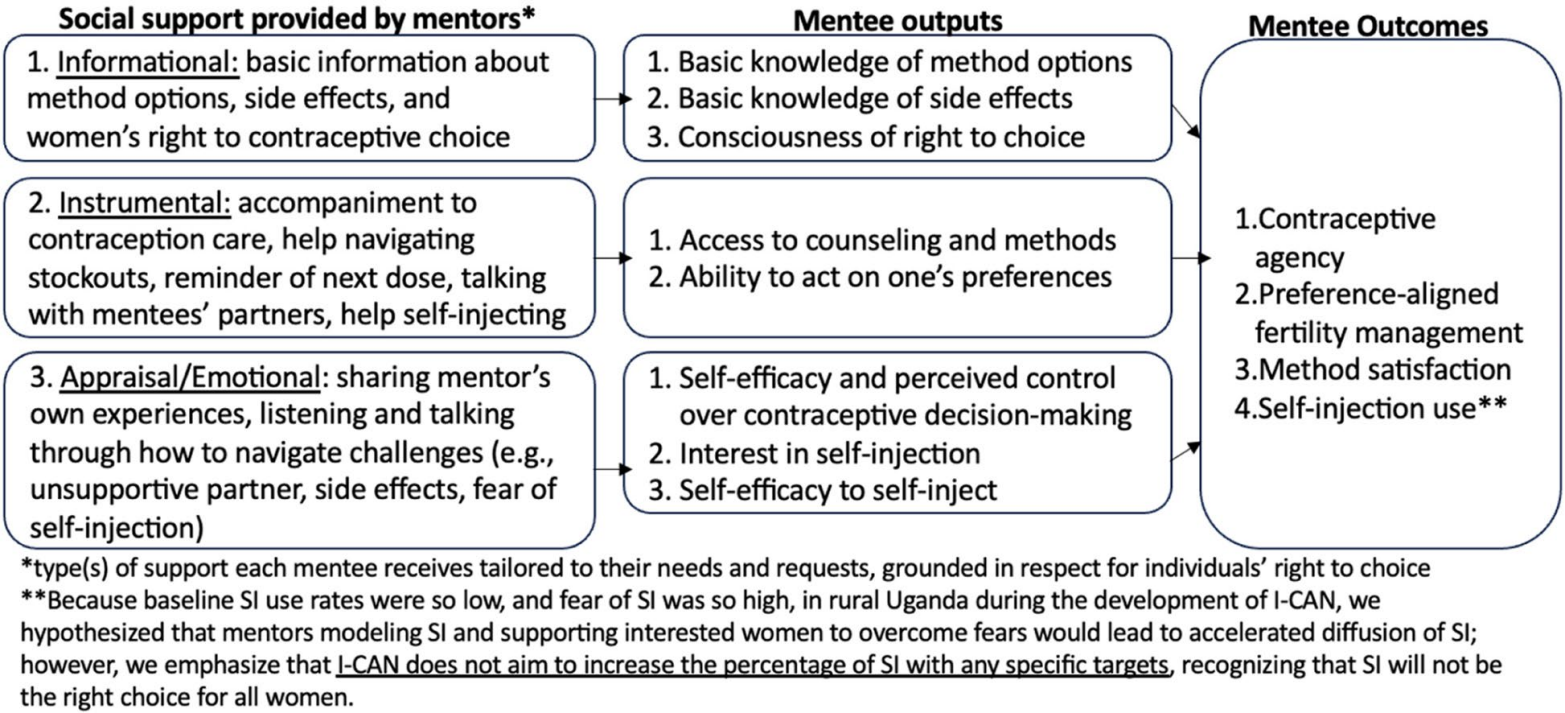
The “I-CAN” Intervention Components and Theory of Action [35, 36, 46, 47]

The dCABs developed simple prototypes of the three final solution ideas via roleplaying, physical material construction, flipchart sketching, and group presentations. Prototypes are rapidly generated depictions of how solutions might be operationalized for implementation that can be quickly tested and refined [44]. Thus, prototypes can take on many modalities and formats. Each dCAB shortlisted a set of prototypes that the research group further built into prototype packages for initial field testing in both districts. As part of this initial prototype refinement process, the research group also developed a theory of action (TOA) [45] informed by the Contraceptive Agency Framework and Diffusion of Innovation Theory. We referenced this TOA (Fig. 2) during prototype refinement and testing to ensure that any changes to the intervention design stayed within the theoretical pathways by which the intervention was hypothesized to work.

In this next round of prototype testing, the research group reintroduced the refined prototypes to the dCABs, who were split into the following groups: 1) SI users, 2) non-SI users (other contraceptive users, non-users, women group leaders), 3) CBO representatives, and 4) village health team members. Facilitators oriented the four testing groups in each district to the prototypes and asked the participants to explain what they understood, liked, and disliked about each prototype, what they would change or add, and how prototypes made them feel about making decisions related to contraceptives.

After using feedback from the first round of testing to refine the prototypes into more complex, detailed versions, the research group tested these “higher fidelity prototypes” using roleplaying with target users while observing the roleplaying and then interviewing the roleplaying actors and viewers after the roleplays. During this second testing round, the research group also engaged the dCAB members who were self-injecting with DMPA-SC to interact with and use the prototypes as if they were offering support to other women. While research assistants observed them, these dCAB members approached their peers at women’s groups, markets, and hair salons to tell them about this intervention and how they could support women with contraceptive decisions. Immediately following these mock interactions, research assistants requested to interview women whom the dCAB members approached to understand their experiences interacting with the pretend peer supporter. The dCAB members also debriefed experiences of ‘playing’ a peer supporter with the research assistants and reflected on what the research assistants reported observing during the live-action prototype testing. We compiled the final testing inputs to refine the solution components into one intervention package.

## Results

### Participants

Women who participated in the in-depth interviews included 19 women aged 20–45 and two aged 15–19. Fifteen were married or living with a partner, while six were single or separated. Most participants (13/21) had two or more children and were primarily engaged in subsistence farming and small-scale businesses selling vegetables. In the six FGDs conducted, five groups consisted of women aged 20 years and above, with the remaining group being young women aged 15 to 19 years old.

### Design outputs

Our design process yielded two outputs that we describe below: 1) insight statements of the needs and opportunities of potential users and 2) a holistic intervention solution.

### User insights

We identified several insights from our design research and prioritized four that reflected those needs, challenges, and opportunities most aligned with our design challenge. Table 2 shows the four insights, specifying associated needs, challenges, opportunities, and an illustrative quote. Additional insights generated from our design process are summarised in Additional File 1. These broadly reflect challenges posed by the formal service delivery system, such as negative experiences of care and women’s living conditions, including overwhelming household responsibilities that hinder access to services and limit women’s ability to act on their contraceptive decisions. The personas, journey maps, and ecosystems reflecting a conglomeration of different target users’ experiences are detailed in Additional File 2.

**Table 2:** Insights defined during the human-centered design process

Insight	Need	Challenge	Illustrative quote	Opportunity
Partner involvement in contraceptive decision-making	Some women need their partners involved in their contraceptive decision-making, and for their partners to be supportive of their contraceptive intentions. However, these women feel unsure how to develop mutual understanding of their contraceptive intentions/preferences with their partners. Underlying this need is women’s fear of conflict if noticeable side effects occur following use without prior involvement of their partner. Concurrently, other women choose to act on their contraceptive intentions despite lack of partner support but, this requires them to conceal their actions/choices	The desire for shared decision-making or covert use forces some women to defer acting on their contraceptive intentions or to abandon their preferred fertility management. Some women fear their partners’ reprisal if their partner uncovers that they are preventing pregnancy	“*I cannot use a method without my husband’s knowledge, if he does not know that I am using a method then this can create violence in my house, there will be trouble between me and him.*” – 23-year-old woman	Women need strategies on how to obtain the desired support of their partners, including how to effectively communicate their fertility preferences or contraception intentions to their partners. Also, women need ways to fulfil their preferences without their partner’s knowledge
Access to contraceptive services and products	Women who choose to use contraception need reliable access to their method of choice but are often unable to get the method they desire due to stockouts and long waiting times at public providers or lack of affordability in private facilities. This is compounded by disrespectful treatment from healthcare workers who question women’s contraceptive decisions and the lack of knowledge of or access to alternate sources	Failure to access the desired method limits women’s ability to act on their preferences to delay or avoid childbirth. This challenge results in fertility misalignment (women using a method that they do not want) and puts women at risk of unplanned pregnancy	“*I went to remove my implant from a different health centre, and the health workers told me to go back to the people that gave it to me in the first place*.” – 31-year-old woman	To support women to act on their contraceptive decisions communicating availability of different contraceptive methods and linking women to alternative access points in their communities are crucial opportunities
Side effects and contraceptive use	Women who experience contraceptive-related side effects, such as prolonged bleeding or irregular menstrual cycles, need support and information to deal with actual and potential side effects. Women feel ill-equipped to deal with side effects, due to inadequate counselling, fear of partner retaliation, and/or lack of an accessible support system. They require equitable access to appropriate alternatives to enable them to achieve their contraceptive desires	Experiencing contraceptive-related side effects and being unable to cope with them contribute to contraceptive discontinuation against women’s preferences, while other women delay or inconsistently use contraceptives	“*I have tried family planning methods like the IUD, implant and pills and I had constant bleeding for a long time! Now I just decided to use the local herbs because some of my friends say that they work for sure*.” – 24-year-old woman	Empathetic support systems at household, community, and facility levels, as well as appropriate counselling on contraception-related side effects, should be accessible to women who choose to use contraception
Contraceptive information sources	Women contemplating contraception use need trustworthy and reliable sources of information to help them make informed choices and act in line with their preferences. Women value learning about pregnancy prevention from their friends or relatives, but also prefer to have information from trained personnel such as community-based and other health workers	Contraceptive information is usually provided in group settings at public healthcare facilities, which may not feel acceptable for some women to safely access the services they need, to share their experiences or to ask personal questions. Also, experiences and information shared by their friends might overwhelm women and without appropriate biomedical guidance from trainer provider, women may fail to act on their contraceptive desires	“*Even if we could meet for family planning discussions at someone’s home, that would be good because of the privacy.*” – 33-year-old woman	There is an opportunity to diversify facility-based contraceptive care with tailored peer-based support for women to learn about and discuss contraceptive-related matters

### Solution Ideas and Final Intervention Design

The 24 ideas generated and shortlisted by dCABs are shown in an Additional table (see Additional file 3). The three leading solution ideas selected by the research group included: 1) identifying messengers, such as experienced contraceptive users, to support women in the community by sharing contraceptive information and lived experiences with contraceptives, including SI; 2) creating safe spaces where women may openly share their contraceptive experiences and help each other to navigate challenges, and 3) communicating where women could find contraceptives they want in their community.

The research group integrated these three ideas into an intervention we coined as *Nsobola* in Lusoga, *An Atwero* in Langi, and *I-CAN* in English**,** after the name of the overall project in which this work was embedded. The “I-CAN” intervention engages experienced SI users (called ‘mentors’) to provide social support for making and acting on contraceptive decisions to women (called ‘mentees’) in their community, including the option to self-inject DMPA-SC if mentees are interested in that option. The high-level intervention design before prototype testing was for mentors to recruit women through community outreach in public settings and for “mentees” to receive social support through one-on-one and group interactions in spaces where they felt safe. Based on social support frameworks, [46] we anticipated support should include informational, instrumental, appraisal, and emotional support mechanisms, whereby mentors would share their experiences with contraception use, provide a listening ear, or link women to formal providers, among other services.

Figure 2 depicts theoretically informed [35, 36] pathways by which we designed I-CAN to influence mentees’ contraceptive agency and self-injection of DMPA-SC via different types of social support. Based on the Contraceptive Agency Framework, [36] we first expect that informational support provided by mentors will increase mentees’ knowledge of contraception and their consciousness of the right to contraceptive choice by 1) sharing essential information about the options available, 2) addressing common concerns and misinformation about side effects (e.g., contraception does not cause infertility, hormonal methods affect women’s bodies differently); and 3) emphasizing that women have a right to decide whether to use contraception (and what method to use) in consultation with whomever they choose. Second, we expect that mentors’ instrumental support will increase mentees’ ability to access contraceptive services and their ability to act on contraceptive choices by 1) accompanying mentees to contraception services (to visit a VHT or health clinic), 2) helping women navigate stockouts (e.g., contacting services to identify available methods), 3) providing reminders for re-dosing periodic methods, 4) talking with mentees’ unsupportive partners, and 5) helping interested women SI. Third, we hypothesize that mentors’ emotional and appraisal support will increase self-efficacy and perceived control over decision-making and self-efficacy to self-inject by 1) sharing mentors’ experiences navigating challenges to contraception and 2) listening and talking through with women how they might navigate barriers (e.g., unsupportive partners, side effects, judgmental healthcare providers, or fear of self-injection). Lastly, based on DOI Theory, we expect that by hearing mentors talk about their self-injection experiences, mentees will be more likely to be interested in trying the option themselves (the *observability* construct), contingent upon the method being provided in a way that allows women to perceive a *relative advantage* of self-injection—e.g., that it offers privacy, convenience, and a sense of control, as identified in prior research [14].

### Prototype development and testing

To build out specific approaches for I-CAN mentorship, we developed four “goals” for prototype testing: identify the ideal mentor and her responsibilities, decide how to raise awareness about the intervention via multiple communication channels, develop concise and acceptable speech for the mentor to approach and introduce herself to potential mentees, and provide topic-specific prompts to guide mentors during their interactions with mentees. The corresponding prototypes to address these goals included 1) a job pamphlet, 2) a program poster, 3) a program pitch for mentors, and 4) reference cards for mentors. Table 3 shows the prototype descriptions and critical results from testing and refining each prototype with the dCAB community members.

**Table 3:** Prototype development, testing, and refinement of the I-CAN solution

**Goal** *(prototype)*	Changes requested during testing round 1*	**Higher fidelity prototype**	**Changes requested during testing round 2**^a^
**1. Identify the ideal mentor and her responsibilities** *(Job pamphlet)*	- add detail on how mentor will engage with formal healthcare system *(potential mentors and VHTs)* - develop a referral card for mentor to refer mentee to VHT *(potential mentors and VHTS)* - outline what mentor’s daily activities entail to help estimate time being a mentor takes *(potential mentors)*	> To make mentor’s job more tangible to potential mentors and mentees, we built a **mentor workflow** to diagram the steps a mentor would typically follow > Each workflow starts with a mentor going to women’s groups or into the community to introduce the program (using the pitches), enrolling women who expressed interest in the program, and providing ongoing, individual support to women	- add opportunities to practice with fellow mentors during a pre-training for mentors to gain confidence to handle different scenarios that may arise during their follow-up support *(potential mentors)* - add that the mentor can demonstrate how to SI and bring women DMPA-SC doses to SI at home *(potential mentees)* - show how a mentor would support a woman without her partner knowing if that’s what she prefers *(potential mentees)*
**2. Decide how to raise awareness about the intervention via multiple communication channels** *(program poster and leaflet)*	- provide alternative ways to learn about the program *(potential mentees)* - include how woman can find mentor on the poster *(potential mentors)*	> To account for women’s different preferences for learning about the program, we designed **leaflets** and **foldable cards** and refined the original poster	- leave a blank space on the materials where mentor can write her name & mobile number if mentee requests and mentor permits *(all groups)*
**3. Develop concise and acceptable speech for mentor to approach and introduce herself to potential mentees** *(Program pitch)*	- prepare pitches for different situations and types of women mentors may meet, such as young women at the hair salon vs older women at the village market *(potential mentors)* - pitches should describe what about the mentor makes her trustworthy *(potential mentees)*	> To engender trust in the mentor, we refined pitches into **shorter pitches** that briefly highlighted mentor’s own contraceptive journey > To capture different target users’ preferred way of meeting a mentor, we adapted the pitches for **formal group settings** (women group meeting), **informal group interactions** (at the borehole), and **1-on-1 encounters**	- mentors should develop their own pitches and practice them during pre-service training *(VHTs)* - prefer doing community outreach one-on-one versus talking to a big gathering; some suggested they may visit groups in pairs *(potential mentors)*
**4. Provide topic-specific prompts to guide mentors during their interactions with mentees** *(Reference card/manual)*	- add more topics that mentees might need mentor’s support with *(potential mentors)* - replace text with visuals where possible *(potential mentors)*	We adapted reference card into **reference manual** and added more topics, including support for SI use, navigating stock-outs, and communicating with unsupportive partner	- practice using the reference manual during their pre-training will help facilitate their job confidence *(potential mentors)* - produce manuals in English and local language in case mentees wish to look at manual during individual visits with mentor *(potential mentees)*

^a^In paratheses is /are the source(s) of the suggested changes

## Discussion

Universal access to sexual and reproductive healthcare services (Sustainable Development Goal 3) requires women to be able to make (and act on) their own decisions regarding contraception [48]. In this paper, we described the application of human-centered design to develop, test, and refine a community-based peer social support intervention to enhance women’s contraceptive agency and support SI use among women who desire it. Below, we discuss critical lessons learned from this design process and reflect on their implications for the future design of contraceptive care programming.

Our participatory and iterative design approach should enhance the likelihood of our intervention’s effectiveness (to be studied in future research) at leveraging tailored social support (appraisal, emotional, informational, and/or instrumental, depending on women’s needs) from mentors to enable women to navigate challenges they may encounter along their contraceptive journeys and to help more women to use SI. By engaging women and other stakeholders in design research, results interpretation, idea generation, and prototyping, we worked to ensure that the resulting intervention centered on users’ needs first and foremost and was highly tailored to the specific context where we developed the intervention (i.e., rural areas of Eastern and Northern Uganda). Community participants appreciated seeing their input reflected in the design outputs throughout the design process. They were eager to see the pilot intervention implemented, highlighting that the HCD approach also helped us lay the groundwork for future buy-in for piloting the intervention in the study sites.

Participatory approaches similar to our HCD approach have been used to develop other contraception care interventions in Uganda, with one study showing promising results [49] and another yet to be evaluated [43]. While these two studies used stakeholder engagement to improve a pre-defined intervention, our approach was unique. It centered on potential users as co-designers in creating an intervention from scratch and represented a promising direction for future intervention development efforts. HCD is increasingly and widely used to develop public health interventions [30]; however, few studies, particularly in sexual and reproductive health, have reported their effectiveness. One study from Kenya indicated an increase in the number of contraceptive users due to a replicated intervention designed using HCD [44]. Another study from Cote d’Ivoire also showed that a youth-designed intervention increased communication about family planning between young and old communities [50]. Another study from Uganda demonstrated the acceptability of an intervention targeting menstrual health, an implementation outcome related to adoption/uptake [31]. Outside SRH, HCD interventions have been reported to improve treatment adherence and retention [51]. The present study focused on the intervention design process. Results of the potential effectiveness of the piloted I-CAN intervention will be published separately.

This collaborative design process challenged our team logistically and philosophically. First, while having two separate design teams (separated by district) encouraged a comprehensive, multi-user-centered process, it also added complexity and time as both teams generated numerous ideas that eventually had to be prioritized and agreed upon amongst diverse sociocultural groups. Second, keeping closely to the HCD approach of using a divergent process for idea generation that values creativity and discourages overly constraining what ideas are on the table led to many initial ideas diverging far from the original design challenge. When we reflected on the initially proposed solution ideas (93 in total), considering their relevance to our specific design challenge of improving contraceptive agency and supporting SI use for interested women, we realized that in “going wide” with the idea generation, the dCABs had generated many ideas that did not directly respond our original design challenge. Specifically, despite orientation on a human rights-based approach to contraception programming and the Contraceptive Agency Framework, we found many initial ideas were overly focused on promoting the *uptake* of contraception over all else.

Continually (re)centering women’s agency, not uptake, related to contraception throughout the design process was crucial and challenging. Encouraging team members to focus on supporting a woman’s contraceptive agency rather than whether a woman uses contraception was not an easy transition. This challenge was likely linked to existing family planning rhetoric familiar in Uganda and much of the world, where, especially during research and analysis phases, many people identified a need to increase contraception use because of what they labeled as a problem of low contraceptive prevalence rates. To address this challenge, we developed the I-CAN theory of action, or as we termed it to the dCABs, ‘our guiding stars.’ We learned that we needed these guiding stars (“1. We want to support women’s contraceptive agency, not necessarily their use of contraception; 2. We have a special focus on self-injectable contraception because it’s so new!”) to more clearly convey our expectations of the final solution to the dCABs to help us ensure the human rights-based approach we had introduced in early sessions with dCABs was ultimately infused into the solutions we collectively generated.

While we necessarily converged our focus in the design process to align with the research question, other insights we discovered (Additional File 1) may be relevant to future design efforts on contraceptive access in similar settings. Furthermore, because the HCD process encouraged us to tailor I-CAN intervention to the local context, additional inquiry as to whether the solution we developed for these rural sub-counties is more broadly relevant elsewhere in Uganda and sub-Saharan Africa would be of value.

Ultimately, I-CAN fills an essential gap in existing contraception services in rural Uganda. Unlike VHTs, whose main function is to provide primary healthcare—including basic contraceptive counseling and provision—in their assigned villages, mentors draw on their lived experience to provide tailored peer support to other women in their community. The proposed mentors’ function complements VHT services by serving as someone who spends more time listening and problem solving with women, and the specific support each mentee receives is tailored to their needs and preferences – thereby individualizing the most impactful pathway(s) of support. We note that our future efforts to operationalize and pilot the I-CAN intervention will necessitate careful attention to training mentors to provide neutral support and respect women’s contraception decisions, acknowledging the potential challenges inherent in coupling a single-method focus (SI) with a broader objective to support contraceptive agency. Training will pay close attention to helping mentors share information about self-injection in a neutral way that raises awareness without biasing women’s choices if they prefer other methods (or no method).

### Strengths and limitations

Our HCD approach included several notable strengths. First, multiple research and design methods helped us understand the context and dynamics surrounding contraceptive decision-making and experiences among our target population. Second, stakeholders’ active participation across all design stages also provided valuable insights to improve the relevance of the intervention. Third, the potential of our intervention was further strengthened by the theoretical underpinnings of our design process – Diffusion of Innovation Theory [35] and Contraceptive Agency Framework [36], which both supported the proposed mechanisms for our intervention’s intended outcomes. Nonetheless, a few limitations exist. Of note, the insights used to develop the intervention were primarily based on women above 20 years of age. With a limited number of adolescents participating in the design research, we may have missed adolescent-specific issues where social support could be applied. However, considering existing knowledge of adolescents' critical reproductive health challenges, such as unfriendly health services and peer influences [52], we believe that our intervention could also benefit this key population, similar to the older women depicted in our personas (Additional File 2). Also, research and design participants mainly came from one sub-county in each district. Thus, insights may not represent other women in the two districts or WRA in different districts of Uganda. Furthermore, most participants were recruited from areas considered rural, and thus, findings may not be generalizable to women living in urban areas. Lastly, male partners of our target population (mentors and mentees) were not engaged during the design process, which may have limited the refinement of the intervention or its components better to suit the lived experiences of the mentees and mentors. Opportunities to engage women’s partners in the intervention design process could include solving for partners’ experiences and preferences related to peer-to-peer approaches delivered in or close to a woman’s residence and what factors might influence their willingness to participate (or to support their partner to participate) in such an intervention.

## Conclusions

Our intervention development process demonstrates that interventions targeting more complex and rights-based outcomes in reproductive health, such as contraceptive agency, are feasible using human-centered design approaches. The engagement of different women from the community and other stakeholders proved instrumental in identifying the intervention's most relevant, acceptable, and viable components. Our participatory and iterative design approach strengthens the likelihood that tailored, peer-based social support will enable women to navigate different challenges along their contraceptive journeys successfully.

## Supplementary Information

Supplementary Material 1.

Supplementary Material 2.

Supplementary Material 3.

## Data Availability

The data supporting the conclusions of this article are included within the article (and its additional files).

## Abbreviations

DMPA-SC: Subcutaneous depot medroxyprogesterone acetate
SI: Self-injection
HCD: Human-centered design
DOI: Diffusion of innovation
WRA: Women of reproductive age
dCAB: Design community advisory board
CBO: Community-based organization
VHTs: Village health teams
IDI: In-depth interview
FGD: Focus group discussion
HMW: How might we
TOA: Theory of action
SRH: Sexual and reproductive health
