# Supplementary material for: Development of a community-based peer-support intervention to improve contraceptive agency and diffuse self-injectable contraception in Uganda: Application of the human-centered design approach
Source: BMC Womens Health. 2025 Mar 10;25(Suppl 1):110. doi: 10.1186/s12905-025-03614-5 (PMC11892169; doi:10.1186/s12905-025-03614-5)
Supplement: Supplementary file 1 — Supplementary Material 1. [file 12905_2025_3614_MOESM1_ESM.pdf]

Additional file 1: Other insight statements generated from the I-CAN human-centered design process

Insight	Frustration	Illustrative quote	Need for the future
**Negative healthcare worker experiences**: Women seek FP information from healthcare workers in health facilities because they believe they are knowledgeable, well trained, and are always ready to provide help.<br><br>They also seek FP information from VHTs because they believe and know that they have been trained and equipped with the right health information	However, many women seeking FP assistance encounter, or hear about mostly negative experiences with healthcare workers who are rude, arrogant and unsupportive. These experiences prohibit some women from seeking FP assistance from health facilities.	"*I went to remove my implant from a different health centre, and the healthcare workers told me to go back to the people that gave it to me in the first place*." **Food vendor**	Positive experiences with healthcare workers are a crucial part of acquiring family planning services.
**Family planning service delivery**: Long waiting queues at the Health Centre coupled with lack of privacy when seeking FP services is a barrier to women's FP use and adoption, especially women who would like their use kept confidential. Furthermore, the fear of being turned away or not being	Inadequate support and confidentiality from HCWs when providing FP services to women are a threat to FP retention and adoption among women. Women who would like their FP use confidential are	"*I went to remove my implant from a different health centre, and the healthcare workers told me to go back to the people that gave it to me in the first place.*" **Food vendor**	HCWs need to provide women with the support and confidentiality that they need when acquiring FP services

helped by health workers (e.g., when removing a method or managing side effects from a method they did not provide in the first place) is also a big concern for women	inconvenienced by the public service delivery of FP information.		
**<u>Existing capacity of healthcare workers</u>**:<br>Diverse roles exist within health facilities, with staff often carrying out multiple tasks (e.g., registering patients, managing triage, treating emergency patients and other walk-in patients at a time) because the hospitals are understaffed.	The heavy workload experienced by staff members limits their ability to provide efficient FP services to patients.	"*You go to the hospital and the nurse is so rude to you! Yet you might find she didn't get enough sleep and has not been paid for the past 3 months.*" **Muslim group leader**	Healthcare workers need support to be able to manage their workloads and at the same time provide quality service to patients seeking family planning services.
**<u>Financial constraints</u>**:<br>Some women, mostly the single and unemployed, are often overwhelmed by other financial responsibilities tied to providing basic needs for their families to make them prioritize family planning. Many of them end up depending on their male partner(s) to meet their financial needs.	These financial constraints experienced by such women hinder their ability to acquire and manage FP methods and associated costs (e.g., transportation to the health facility and treating side effects like excessive bleeding), and may result in them abandoning use if their	"*Sometimes I wish my mother was still alive so that at least I would have support with the children and I could go and look for work.*" **Restaurant owner**<br><br>"*If my client does not want to use condoms, I just have to accept because otherwise I will not get paid.*" **Sex worker**	These women need to be equipped<br>to efficiently plan and generate the<br>necessary finances independently,<br>in order to have the freedom of choice of a FP method that suits their lifestyles

	male partners do not support FP use.		
**<u>Cultural influences</u>**:<br>Vulnerable groups of women like sex workers and single mothers experience stigma within their communities tied to their identity Some mothers and older women are not comfortable having conversations on sex and family planning with their daughters and young girls. This is because these conversations are usually handled by aunties and other relatives.	This hinders them from acquiring FP services that they need due to fear of being shunned or ridiculed.<br><br>This leaves many young girls frustrated as they try to find alternative sources of information.	"*I know what they say about me that's why I don't like going to the health entre….they say that I am shameless. Even the nurse was so rude to me last time and refused to give me my Sayana dose. For me, I know it's because I separated from my husband so they don't expect me to have sex*". **Single mother (Divorcee)**<br><br>"*I know what they say about me that's why I don't like going to the health centre….they say that I am shameless. Even the nurse was so rude to me last time and refused to give me my Sayana dose. For me, I know it's because I am a sex worker*". **Sex worker**	Single women should be able to access FP services that they need despite their marital status.<br><br>Cultural sensitivity tied to discussing topics around sex and family planning needs to be addressed in order to increase uptake and encourage information sharing among women of reproductive age.<br>A culture that encourages conversations around FP and SRH needs to be fostered among women of reproductive age.<br><br>Healthcare workers need support to be able to manage their workloads and at the same time provide quality service to patients seeking family planning services.

**Women responsibilities**: Juggling between house chores, jobs/ work and looking after children becomes difficult for women if they do not have extra help. In the event that the help is unavailable, women carry their children everywhere that they have to go, which is quite tasking.	Women are too busy to remember their FP refill and follow up days.<br>Some of them who do remember are heavily engrossed in their work to spare time for FP services	"*Men are never home. Women are too busy doing very many chores all the time to even remember to go and get their next dose of FP like sayana press and pills*." **Married woman, 2 children**	Due to the fact women are already overwhelmed with other tasks, they need recurring external help with following up on their FP use
