# Supplementary material for: Development of a community-based peer-support intervention to improve contraceptive agency and diffuse self-injectable contraception in Uganda: Application of the human-centered design approach
Source: BMC Womens Health. 2025 Mar 10;25(Suppl 1):110. doi: 10.1186/s12905-025-03614-5 (PMC11892169; doi:10.1186/s12905-025-03614-5)
Supplement: Supplementary file 2 — Supplementary Material 2. [file 12905_2025_3614_MOESM2_ESM.pdf]

Additional file 2: Personas, Journey Maps and Ecosystems

Personas

A) Vanessa persona

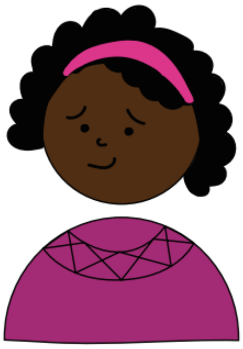

"...one of these days I will get an opportunity and shift to a big city.."

Name: Vanessa
Status: Unmarried
Age: 20 years
Parity: O children
Occupation: A student
FP method: Non user intending to use either pills or an injectable (not sure of what type of injectable).

Vanessa works as a shopkeeper in her parent's shop in town. She lives with her aunt near the trading centre. She is sexually active with her boyfriend and has always wanted to get a FP method but is afraid of being judged by hospital staff and people in the community because she is young and unmarried.

Her boyfriend is indifferent to her use of a FP method.

GOALS:
- To be employed in a bank in the city.
- Needs to find a long acting free FP method that works for her, so she can focus on her studies.
- To find a reliable and accessible source of FP information.

MOTIVATIONS
- To make enough money to complete her university studies
- To prevent unplanned pregnancy

RESOURCES
- University student-learning accounting
- Tech-savy
- Income: ugx 60,000 per

FRUSTRATIONS:
- Little knowledge on FP
- Community pressures to get married
- Would like to use FP but is uncertain of which type because of the side effects she has heard about.
- Limited access to life mentorship (career, health and wellness, financial).

B) Milly persona

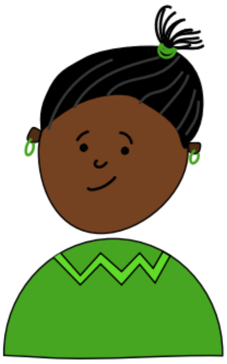

"...my husband is a very respected man in our community, everyone listens to him!"

Name: Milly
Status: Married
Age: 27 years
Parity: 2 children
Occupation: A subsistence farmer
FP method: Non user.

Milly is proud of her garden. She takes good care of it, almost like it is part of her family. Every thursday and monday she sells excess food at the market day, but also has a small kiosk outside her home for everyday buyers.

She once brought up the idea of FP to her husband and he rejected it. She always hears him telling his friends that he is against FP. He has heard mostly negative things about it and is not ready to be the next victim.

GOALS:
- To be a large scale commercial farmer in her village
- To convince her husband to support their use of a FP method.
- To space her children

MOTIVATIONS
- Tadeo, a commercial farmer in the village who offers her farming and business advice.
- Sarah a close friend, who provides a lot of emotional and psychological support.

RESOURCES
- Feature phone
- Income- unpredictable about ugx 5000 a day

FRUSTRATIONS:
- Her husband is not in support of Family planning use
- She does not have enough money to cater for some of her personal needs.
- She feels unsupported in her community to seek FP because she can't go against her husband's wishes, as he is a well known and respected man in the community.

C) Bridget persona

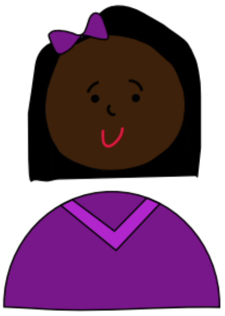

"...I need to make enough money to take care of my children. Sometimes the bar doesn't make enough money"

Name: Bridget
Status: Single
Age: 28 years
Parity: 2 children
Occupation: Bar owner, sex worker
FP method: Condoms, local remedies.

Bridget is a single mother of 2 and runs a bar business but is also a well known sex worker in the community.

She has used multiple contraceptives including the IUD, injectable, pills, and local herbs. She is HIV +ve and mainly uses condoms and a 6 month local remedy.

GOALS:
- To have a reliable and accessible FP method
- To be financially independent and support her family.

MOTIVATIONS
- Her mother who usually helps her with her children.
- To provide for her children's needs
- Peer support from the savings group with fellow sex workers.

RESOURCES
- Feature phone
- Income- unpredictable about ugx 15000 a day

FRUSTRATIONS:
- A lot of unreliable information on FP options
- Prolonged bleeding from using FP
- Stigma from community
- Inadequate income to support her family's needs.

Journey maps

A) Vanessa journey map

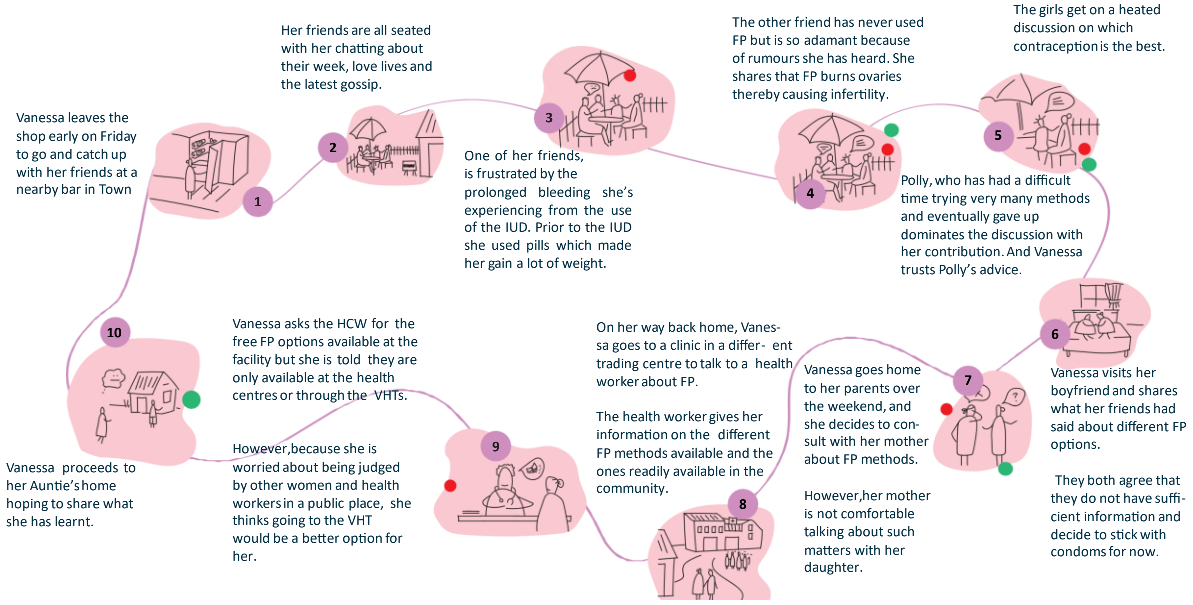

Pain points **Opportunity points**

7, 10 Cultivating a culture that encourages SRH and FP talks between older women and young girls

4, 5 Equipping members of peer groups with verified FP information.

B) Milly journey map

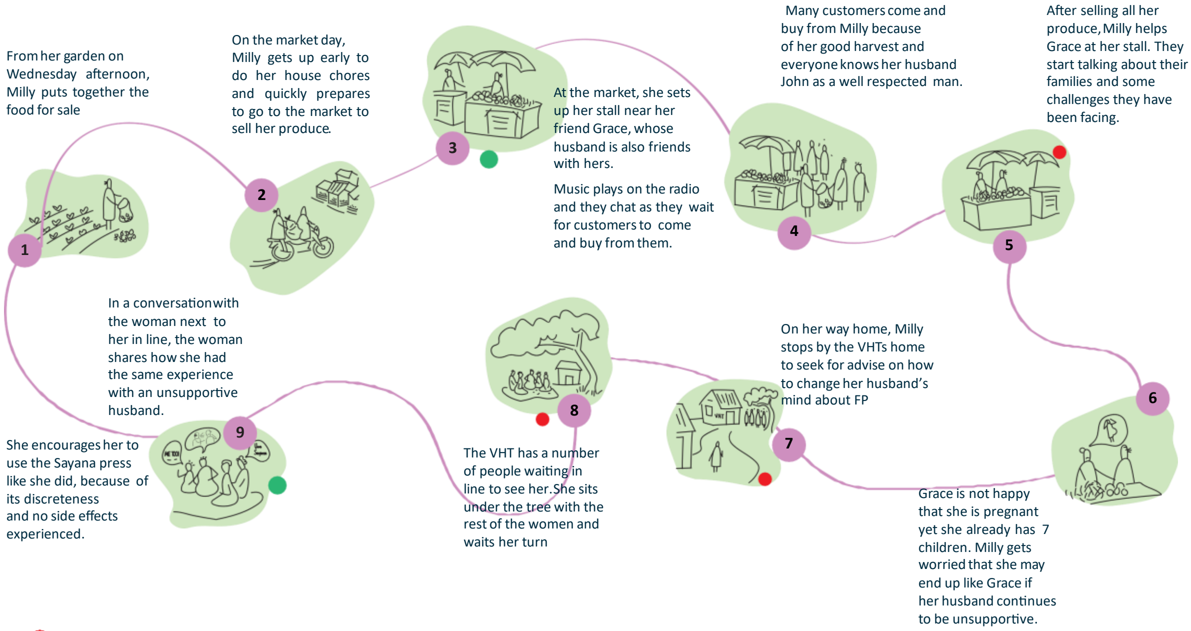

Pain points **Opportunity points**

2 Sharing FP information in locations where large crowds gather.

9 Equipping members of peer groups with verified FP information.

C) Bridget journey map

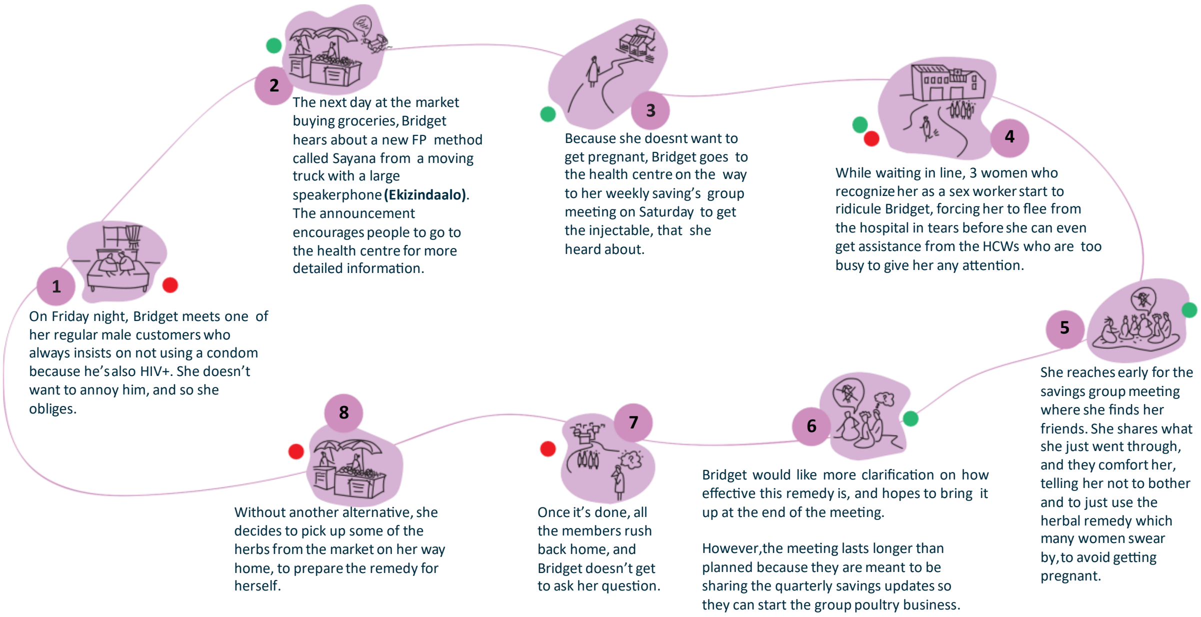

Pain points **Opportunity points**

2 More information can be shared using Ebizindaalo."

4 Sensitising community members on importance of FP for vulnerable groups.

5 Equipping members of peer groups with verified FP information.

6 FP discussions with trained experts to provide factual and relevant information.

Ecosystem maps

A) Vanessa ecosystem map

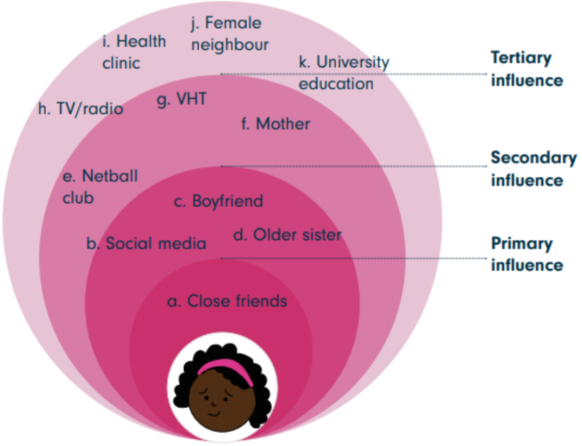

Ecosystem map

LEGEND

a. Close friends
Vanessa's friends usually share their experiences and views on different FP methods.

b. Social media
She gets some information on FP from whatsapp messages and facebook

c. Boyfriend
Vanessa's boyfriend is in support of her using a FP method

d. Older sister
Vanessa's older sister is a trusted source of information. She often gets advice from her.

e. Netball club
Girls at the netball club discuss very many topics and issues concerning them including FP.

f. Mother
She always asks her mother about FP but her mother is not comfortable discussing sex related topics with her

g. VHT
The VHT offers a personal FP service to Vanessa's sister. Vanessa would consider her too for FP services.

h. TV/Radio
Occasionally on radio, advertisements on where to go for more FP information is shared.

i. Health clinic
Vanessa fears judgement from health workers and other women when at the health clinic because she is not married.

j. Female neighbour
Issues on SRH, and FP are sometimes discussed with neighbours.

k. University education
She wants to complete her university education before she has any children.

B) Milly ecosystem map

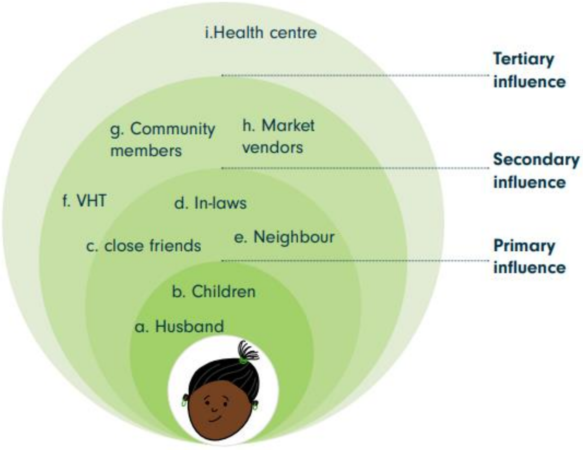

Ecosystem map

LEGEND

a. Husband
Milly cannot use FP without the support of her husband

b. Children
Milly does not want to have any more children.

c. Close friends
Milly trusts FP information received from her close friends.

d. In-laws
Milly's in-laws want Milly to have many children in order to expand their lineage.

e. Neighbour
Milly's neighbour always shares her experience with FP use with Milly.

f. VHT
She gets information on FP from the VHT everytime she goes there to meet her.

g. Community members
She fear community's reaction to her use of FP when her husband, a public figure is against it.

h. Market vendors
There are health talks and FP discussions among market vendors as they wait for customers.

i. Health clinic
She occasionally sits and listens to information on FP shared at the HC

C) Bridget ecosystem map

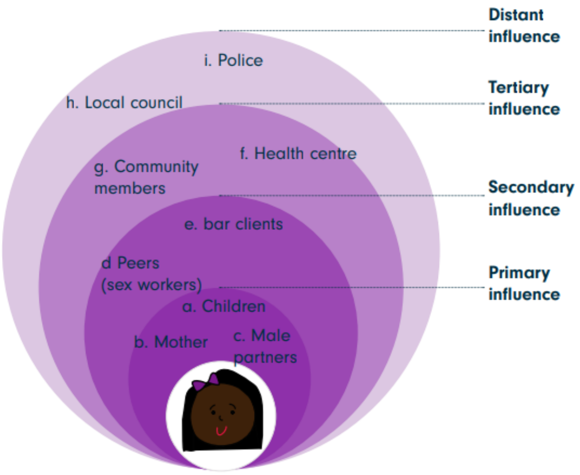

Ecosystem map

LEGEND

a. Children
She wants to be able to provide the basic needs for her 2 children.

b. Peers (sex workers)
Bridget and her fellow sex workers support each other whenever they might be facing a challenge, and also share FP experience with each other.

c. Male partners
Some of Bridget's male partners do not want her to use condoms.

d. Community members
She experiences stigma from the community that discourages her from going to the health centre to seek FP services.

e. Health centre
The health centre is a convenient location for her to get FP services when she goes for HIV treatment.
