# Supplementary material for: Development of a community-based peer-support intervention to improve contraceptive agency and diffuse self-injectable contraception in Uganda: Application of the human-centered design approach
Source: BMC Womens Health. 2025 Mar 10;25(Suppl 1):110. doi: 10.1186/s12905-025-03614-5 (PMC11892169; doi:10.1186/s12905-025-03614-5)

Additional File 3: Selection of solution ideas generated during idea generation phase

HMW statement	Initial solution ideas
'How Might We' communicate availability of contraceptive methods, including self-injectables, at community access points to ensure all women have consistent access to contraception?	1. Community groups should have knowledge and info about the different contraceptive methods<br>2. We should work with community structures like VHTs, social workers, etc<br>3. We should work with group leaders and group members to transfer contraceptive information<br>4. Use areas where community members gather most, like markets, to pass info about FP<br>5. Put up posters around the community and gathering places like borehole area<br>6. Using radio adverts and schools to update on what's available and where re contraceptives<br>7. Using telephones to send information and messages regarding contraception availability at nearby clinics<br>8. Using of elderly gatherings as a way to share FP information<br>9. Use workshops and trainings
'How Might We' equip social groups with factual and relevant contraceptive information to support women in making and acting on contraceptive decisions, including trying self-injection?	1. Designing posters or pamphlets (passive information sharing)<br>2. Using community speakers<br>3. Having a safe place where people can freely share info<br>4. Using FP users – (Peer-to-peer FP champions, incentivized to support women in choosing FP and also supporting SI users in self-injecting<br>5. User stakeholders/influencers in the community)<br>6. Use different play activities like drama<br>7. Design t-shirts, bags with printed info about FP<br>8. Use talk shows on radios about the importance and effects of FP<br>9. Use the internet to share info about FP<br>10. Distribute bicycles to mobilisers<br>11. Use mobile outreach<br>12. Community-oriented dialogues and events to share different perspectives on FP<br>13. Community skits/game nights sharing different perspectives of FP<br>14. Video series on different FP journey touchpoints, group conversation on each video<br>15. Curated FP info and conversation guide to share with women's groups

Refined solution 1: from the original Miro board

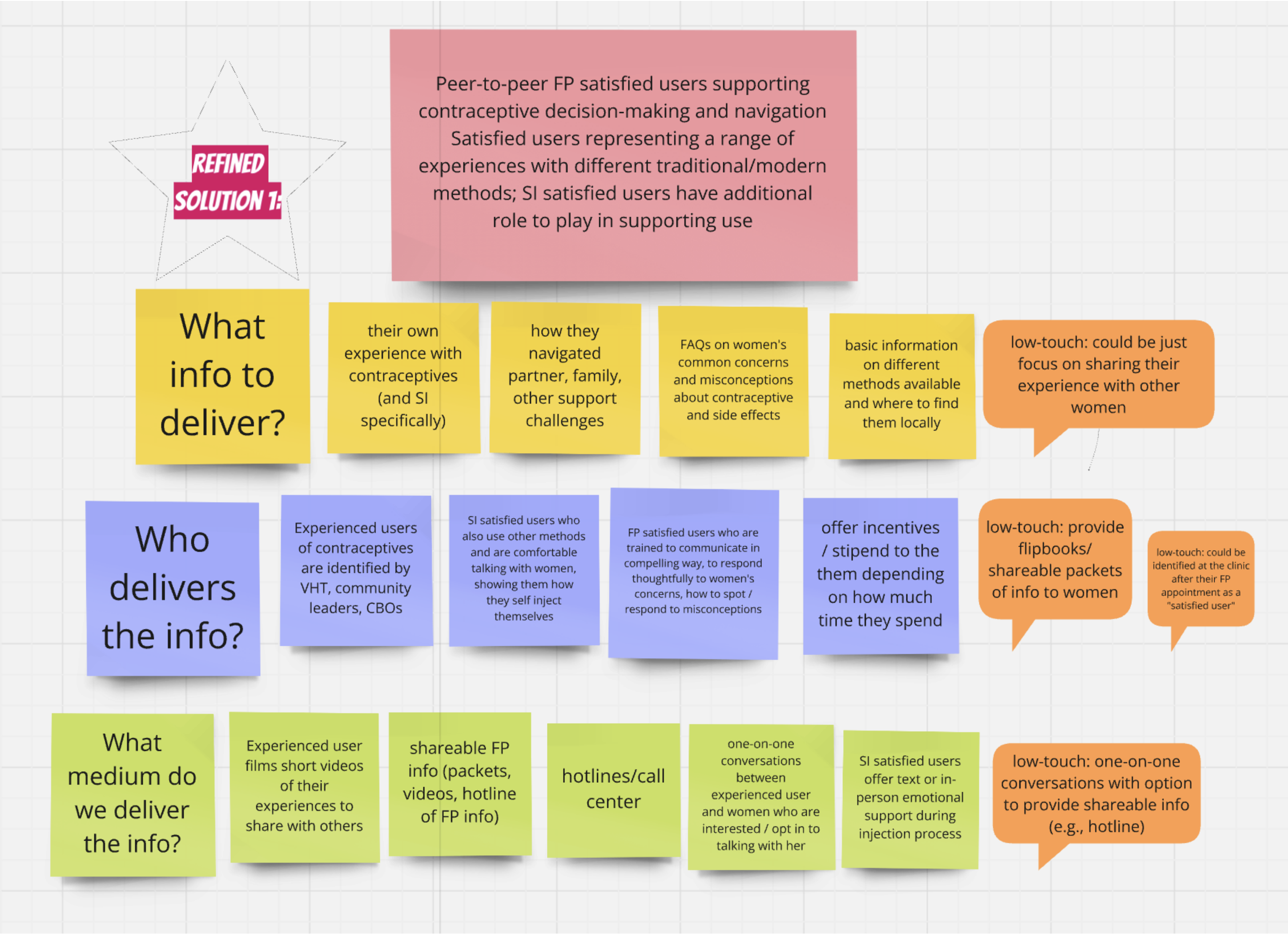

Refined solution 2: from the original Miro board

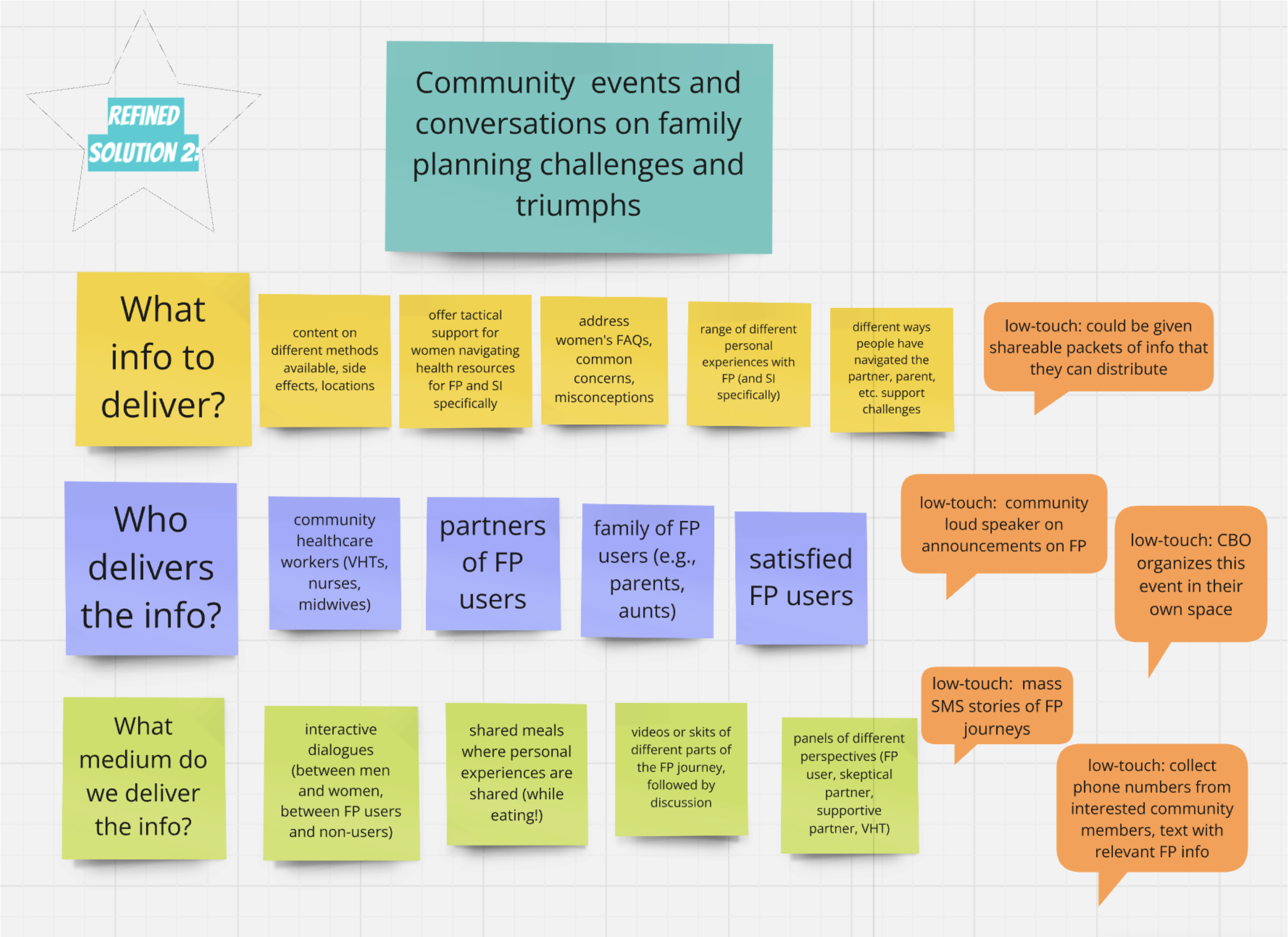

Supplement: Supplementary file 3 — Supplementary Material 3. [file 12905_2025_3614_MOESM3_ESM.pdf]
